# Shear-induced microstructures and dynamics processes of phospholipid cylinders in solutions

**DOI:** 10.1038/s41598-019-51933-z

**Published:** 2019-10-28

**Authors:** Yue Shan, Xiaowei Qiang, Jianzhu Ye, Xianghong Wang, Linli He, Shiben Li

**Affiliations:** 1 Department of Physics, Wenzhou University, Wenzhou, Zhejiang 325035 China; 2 Department of Physics, Wenzhou Vocational and Technical College, Wenzhou, Zhejiang 325035 China

**Keywords:** Biochemistry, Biophysics, Chemical biology

## Abstract

Shear-induced microstructures and their corresponding dynamic processes are investigated for phospholipid cylinders in aqueous solution by dissipative particle dynamic simulation. Various phospholipid cylinders with cross-sections, which are formed under shear-free flow, are selected to examine the effects of shear flow on their structures and dynamic processes. Shear flow induces the transition from cylinders into vesicles at weak rate and the transition into vesicle–lamella mixtures with increased shear rate and lamella structures at the strong shear rate. Then, the average radius of gyration and shape factors of the polymer chains in the dynamic processes are discussed in detail. Results show that shear flow causes the structure of the polymer chains to be elongated along the shear direction, and the configuration of the polymer chain can be rapidly transformed into an ellipsoid structure under strong shear.

## Introduction

Phospholipid molecule is an important category of biomolecules in biological organisations. Given that phospholipid molecules commonly contain one hydrophilic functional group and two hydrophobic fatty acid groups, which are amphiphilic in water environments, the phospholipid molecules can spontaneously self-organise into a rich variety of microstructures with various symmetries, such as lamellae, cylinders, spheres and other complex network structures^1^. These lipid-based liquid crystals can serve as carriers of antimicrobial peptides in a controlled manner to match efficient treatments, such as cubic and hexagonal structures^2–4^. Microstructures, such as lamellar structures, play a basic role in the vital functions of cells and organisms. For example, membranes with bilayered lamella structures in prokaryotic and eukaryotic cells do not only encapsulate and protect the interior of cells but also provide the molecular organisation of vital cellular processes^5^. We are also aware that a series of bicontinuous cubic phases can be formed for monoelaidin (ME), monovaccenin (MV), monoolein (MO) and monolinolein (ML) in water environments^1,6,7^. For example, cubic phases with Ia3d, Pn3m and Im3m symmetries have been reported in complex structures, such as in ME–water systems^6^, and the phase transition between cubic diamond and primitive phases was also investigated in MO–water systems^7^. However, ME, MV, MO and ML, as the other types of lipid categories, have one hydrophilic group and one hydrophobic group, which differs from phospholipids with two hydrophobic groups.

Several factors, such as concentrations, chain lengths and interactions of phospholipids, determine the self-assembly structures of phospholipid molecules in aqueous solutions. For example, X-ray and terahertz spectroscopy revealed that water content determines the phase transition between lamellae and inverted hexagonal phases in phospholipid–water mixtures^8^. Molecular dynamic (MD) simulations have predicted that water operates cooperatively with structural changes for the phospholipid membrane^9^. Lamellar structures have also been investigated in aqueous solutions by MD, in which detailed features were captured in membranes with molecular resolutions^10,11^. Dissipative particle dynamic (DPD) simulation suggests that vesicle structures can be observed in the simple model of phospholipid–water mixtures when chain lengths and amphiphilicity are suitable^12^. Recently, we carried out a DPD simulation of the self-assembly of phospholipid molecules in aqueous solutions and found that the phospholipid polymer chains can self-assemble into different structures in aqueous solutions when phospholipid chain lengths and concentrations are changed^13^. DPD simulations can predict a series of microstructures, including bilayer membranes, perforated bilayer membranes, micelles and vesicles. Under such shear-free conditions, structural shapes are dependent on several factors, including head-to-tail ratio of chain lengths and phospholipid concentrations^13^. When the phospholipid concentration is increased to a suitable value, the cylindrical network structure can be observed (Fig. S1 in the Supplementary Materials), and this condition is independent of chain lengths in the concentrated solutions^14^.

Applying shear flow provides an external force that can induce the phase transitions of phospholipid polymers in solutions instead of varying the inner polymer parameters, such as chain lengths; as a result, corresponding dynamic processes are introduced into the microstructures^15,16^. Phospholipid membranes, a basic bio-structure in cells exposed to hydrodynamic fluid flows, under shear flows have been increasingly investigated, such as hemoglobin flowing in the blood. Shear flows not only redistribute the molecular locations in membranes but also induce the structural changes of membranes and the corresponding dynamic processes caused by hydrodynamic forces^17–22^. For example, external liquid flows result in hydrodynamic forces on molecules on the phospholipid bilayer that causes them to move in the direction of the shear flow and leads to the local concentration of molecules change. Quantitative studies on the magnitude of shear forces have been carried out^21^. MD simulation predicted that shear force leads to the rotation and re-alignment of molecules in phospholipid membranes; these phenomena result in structural undulations that propagate in the perpendicular direction when the shear rate exceeds a critical value^19^. However, layer sliding can occur instead of undulation in phospholipid bilayers under shear flow, and the intermonolayer slip depends on the velocity profile^20^. Dynamic processes, such as changes in system energy and changes in the radius gyration of structures, have also been investigated. For example, the system energy and radius of gyration have been calculated by MD simulation for phospholipid membranes; the results showed that system energy tends to be stable, and gyration radius increases when the shear increases^14^. Shear flows also induce various microstructures, not just lamellar structures, for block copolymers in solutions^23^. In particular, shear flows can induce structural changes in phospholipid vesicles whose shapes are similar to red blood cells, which become exposed to hydrodynamic fluids and cause structural changes^24–33^. Additionally, shear flows can induce shape changes in entire phospholipid vesicles along with domain diffusions and round domains^24^; the deformation of phospholipid vesicles into steady ellipsoidal shapes in constant orientations under simple linear shear flow has been observed by phase contrast microscopy^28^.

Many experiments and simulations have been performed to determine the effects of shears on phospholipid membranes and vesicles. However, the shear effects on phospholipid cylinders still need to be explored. In the current study, we investigate the shear-induced microstructures and the dynamic processes of phospholipid cylinders in solutions by using the DPD and CG model, which is useful in simulating complex fluids and soft matters^34–37^. We focus on the influence of shear flow on phospholipid cylinders with various cross-sections in different concentrations. By using a variety of initial state inputs, the energy of the formed equilibrium structure can be compared, and the simulation method of the stable structure can be selected. Different shear rates are considered to examine the effects on the microstructures and their corresponding dynamic processes. The rest of the paper is structured as follows. Section II describes the model and the method. Section III presents the results and discussion. Section IV presents the summary.

## Model and Method

### Phospholipid model

**Figure 1:**
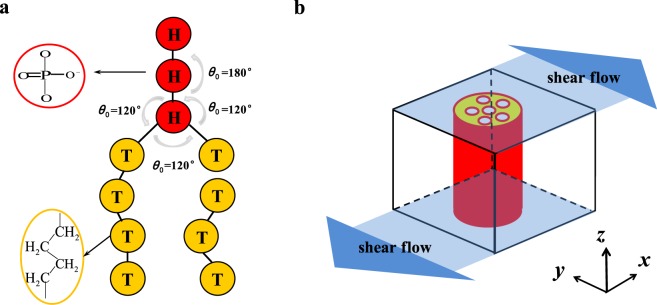
Schematic view of the computational model. (**a**) Coarse-grained lipid model consisting of the hydrophilic beads (red: the upper three particles), and the hydrophobic beads (yellow: the bottom double tail particles). (**b**) Illustration of phospholipid pores under the shear flow.

We coarse-grain a small group of atoms into a single bead in the current phospholipid model. The phospholipid molecule is modelled with two-tail linear chains and one head linear chain, which has been extensively used in the previous studies, as shown in Fig. 1a ^38^. In the phospholipid model, the hydrophilic heads (H) and hydrophobic tails (T) are represented by red and yellow beads, respectively. In order to connect two consecutive beads, we used an additional elastic harmonic force in the model. And this elastic harmonic force can be expressed as follows:

1 $${{\bf{F}}}_{ij}={k}_{s}(1-\frac{{r}_{ij}}{{r}_{s}}){\hat{{\bf{r}}}}_{ij}$$

where the *i*th and *j*th beads are two consecutive beads, and *k*_*s*_ represents the spring constant and *r*_*s*_ is the equilibrium bond length between the two beads, whereas *r*_*ij*_ denotes the distance between *i*th and *j*th beads, with the unit vector ${\hat{{\bf{r}}}}_{ij}$. We take *k*_*s*_ = 100.0 and *r*_*s*_ = 0.7 *r*_*c*_, which are similar to those in the previous studies^39,40^. Here, we set *r*_*c*_ is the cutoff radius in the DPD simulations. An additional force caused by the harmonic constraint is applied onto two the consecutive bonds to achieve the bending resistance of the chains and is given as follows:

2 $${{\bf{F}}}^{\theta }=-\,\nabla [{k}_{\theta }{(\theta -{\theta }_{0})}^{2}]$$

where *k*_*θ*_, *θ* and *θ*_0_ are the bending constant, inclination angle and equilibrium angle, respectively. We take *θ*_0_ = *π* for three consecutive T beads and *θ*_0_ = 2/3*π* for the three consecutive beads at the connective point between the T and H, as shown in Fig. 1a, which are similar to those of the model used in our previous works^13,41^. In this study, we fix *N*_HB_ = 3 and *N*_TB_ = 4 for H and T, respectively. The model phospholipids are integrated into the aqueous solution, in which the water molecules are also coarse-grained. Then, we define the phospholipid concentration as follows:

3 $${\varphi }_{{\rm{P}}}=\frac{{N}_{{\rm{HB}}}+2{N}_{{\rm{TB}}}}{{N}_{{\rm{HB}}}+2{N}_{{\rm{TB}}}+{N}_{{\rm{WB}}}}$$

In the current simulations, we focus on the phospholipid concentrations of *ϕ*_P_ = 0.35, 0.40, 0.45, 0.50. The phospholipid molecules assemble into cylinders with porous cross-sections under the shear-free condition.

### Shear flow

**Figure 2:**
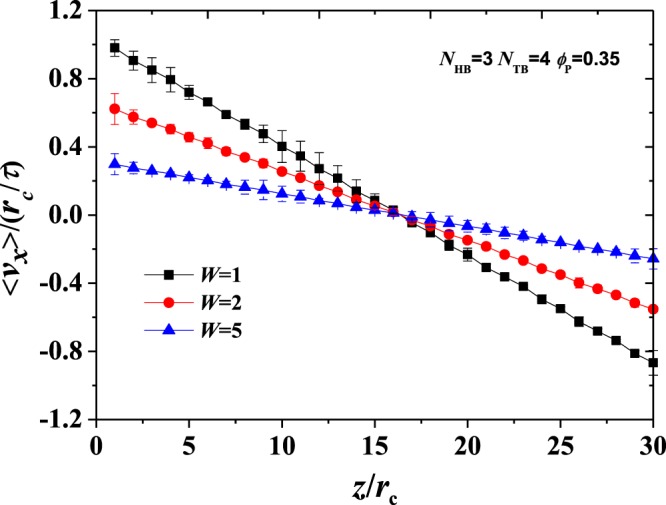
Illustration for the reverse non-equilibrium shearing. An example of velocity profile at three shear rates in the simulation box with a selected set of parameter *ϕ*_P_ = 0.35, *N*_HB_ = 3 and *N*_TB_ = 4.

In linear response theory, a flux relates to a thermodynamic force or field. In the isotropic fluid, the flux has a linear relationship with the corresponding field with a transport coefficient. In non-equilibrium MD, the field is applied, whilst the flux is measured^42,43^. By contrast, in reverse non-equilibrium MD, the momentum flux is introduced, whilst the force or field is measured^18,44,45^. We have successfully applied this reverse non-equilibrium approach in the DPD simulations^14^. Here, we only briefly discuss this approach as follows. The momentum flux is introduced into the system in an unphysical way. Specifically, the periodic simulation box with box length *L*_*z*_ is divided into several slabs in the *z*-direction. The beads inside the slabs at *z* = 0 and *z* = *L*_*z*_ are propelled in two opposite *x*-directions, respectively, as shown in Fig. 1b. The particles can then obtain the largest momentum in the opposite directions in the two slabs. In proving that such shearing is reasonable, the velocity <*v*_*x*_> distribution in this method under the three shear rates is used to roughly satisfy the linear relationship, as shown in Fig. 2 ^44^. Then, the momentum component *p*_*x*_ is exchanged by executing the flip algorithm at regular time intervals. Thus, the momentum flux *j*_*z*_(*p*_*x*_) can be calculated by

4 $${j}_{z}({p}_{x})=\frac{{p}_{x}}{2t{L}_{x}{L}_{y}}$$

where *t* is the time length of the two swaps, and factor 2 arises because of the periodicity of the simulation box with *L*_*x*_ and *L*_*y*_ in the *x*- and *y*-directions, respectively. During simulation, we perform the velocity swap every *W* time steps to satisfy *t* = *W*Δ*t*. We can adjust the time lapse *W*Δ*t* between the two velocity swaps to control the momentum flux *j*_*z*_(*p*_*x*_). In this study, we apply the steady shear flows, in which the velocity distributes linearly in the *z*-direction. In the steady state, the rate of momentum transferred by the momentum swaps is equal to the momentum flowing back through the fluid by friction. In this manner, the unphysical momentum swap conserves the linear momentum and the kinetic energy of the system as a whole because bead positions are unchanged. Moreover, shear rate can be calculated from $\dot{\gamma }=\partial {v}_{x}/\partial z$ with the collinear momentum flux in this method. On this basis, we can obtain the shear rates $\dot{\gamma }/{\tau }^{-1}$ = 0.02, 0.04, 0.06 by setting *W* at 5, 2 and 1, respectively.

### DPD method

In order to simulate the hydrodynamic behaviour of complex fluids and soft matters, we often use the DPD method. In DPD simulations, several pairs of forces are introduced to utilise beads that are coarse-grained from a small group of atoms. The main formulations about the DPD simulation are similar to those used in complex fluids and soft matters^35,39,46–48^. During DPD simulation there are three forces acting on the *i*th bead: a conservation force (${{\bf{F}}}_{ij}^{C}$) from a potential, a dissipative force (${{\bf{F}}}_{ij}^{D}$) that attempts to reduce radial velocity differences between the particles and a random force (${{\bf{F}}}_{ij}^{R}$) that represents stochastic impulse. We can expressed the total forces on the ith bead as follow:

5 $${{\bf{F}}}_{i}=\sum _{i\ne j}({{\bf{F}}}_{ij}^{C}+{{\bf{F}}}_{ij}^{D}+{{\bf{F}}}_{ij}^{R})=\sum _{i\ne j}[{a}_{ij}w({r}_{ij}){\hat{{\bf{r}}}}_{ij}-\gamma {w}^{2}({r}_{ij})({\hat{{\bf{r}}}}_{ij}\cdot {{\bf{v}}}_{ij}){\hat{{\bf{r}}}}_{ij}+\sigma w({r}_{ij}){\zeta }_{ij}\Delta {t}^{-1/2}{\hat{{\bf{r}}}}_{ij}]$$

where *a*_*ij*_ is the maximum repulsive force between the *i*th and *j*th beads, and *r*_*ij*_ is the distance between the *i*th and *j*th beads and *v*_*ij*_ denote the relative velocity between the *i*th and *j*th beads, with the unit vector (${\hat{{\bf{r}}}}_{ij}$). We take *a*_*ij*_ = 200 for the hydrophilic type of beads and *a*_*ij*_ = 50 for the hydrophobic types. The validity of fluctuation–dissipation theorem requires two parameters, namely, $\dot{\gamma }$ and *σ*, to be linked by ${\sigma }^{2}=2\dot{\gamma }{k}_{{\rm{B}}}T$, where *k*_B_ is the Boltzmann constant and *T* is the system temperature^49^. We used *σ* = 3.0 in simulation which is usually the standard valued before^39^. *ζ*_*ij*_ represents a Gaussian distribution of zero mean and unit variance, and the weight function *w* (*r*_*ij*_) can be expressed as

6 $$w({r}_{ij})=\{\begin{array}{cc}1-\frac{{r}_{ij}}{{r}_{{\rm{c}}}} & {r}_{ij} < {r}_{c}\\ 0 & {r}_{ij} > {r}_{c}\end{array}$$

where *r*_*c*_ is the cutoff radius.

### Simulation parameters

The length, time and energy in DPD simulation can be normalised by several parameters. In particular, energy can be normalised by *k*_*B*_*T*, whilst length is normalised by the cutoff radius *r*_c_ in accordance with *r*_c_ = (*ρV*_b_)^1/3^, where *V*_*b*_ is the volume of one DPD bead, and *ρ* is the bead density. Here, we set *ρ* = 3 in the simulation, and the DPD bead usually has an assumed volume of *V*b = 0.03 nm^3^, which leads to a value of *r*_c_ = 1.0 *nm*^50,51^. Time unit *τ* is defined as $\tau ={r}_{{\rm{c}}}\sqrt{m/{k}_{{\rm{B}}}T}$, where *m* is the bead mass. The time unit can be deduced by considering the in-plane diffusion constant of the lipid in the experiment^52,53^. The movement of the DPD beads is driven by DPD forces based on Newton equations, which are integrated by a modified version of the velocity Verlet algorithm with the time step of Δ*t* = 0.01*τ*^47,54^. All simulations are performed on an NVT ensemble by using LAMMPS. The simulation is performed in a box set to *V* = *L* × *L* × *L*, we set all three directions with periodic boundary conditions^34^. Additional simulations are carried out by varying the box sizes to avoid the finite size effect, and the box sizes are optimised to *L* = 30*r*_c_ ^55^. After inputting different initial states, the self-assembly is performed, the energy value in the equilibrium state is compared and the structure with the smaller energy value is selected as the final stable structure. According to the comparison of simulated energy, the final stable state can be achieved after 200000 DPD time steps, which is similar to those in the previous works^56,57^.

## Results and Discussion

**Figure 3:**
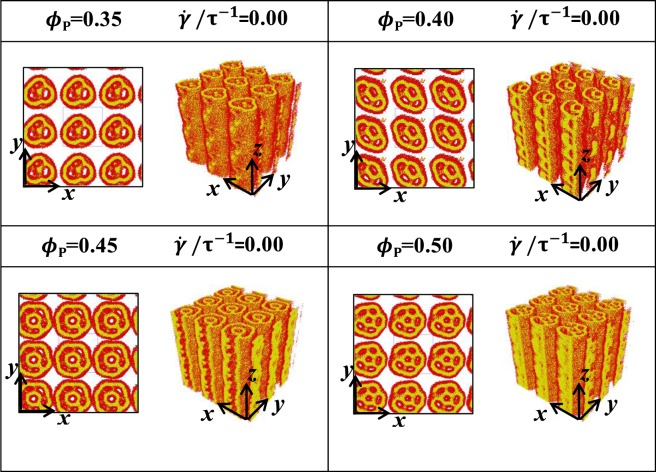
Microstructures of phospholipid pores with *N*_HB_ = 3 and *N*_TB_ = 4. The shear flows are applied perpendicular to the axes of pores, i.e., the z directions. The microstructures are arranged as functions of phospholipid concentrations *ϕ*_P_ = 0.35, 0.40, 0.45, 0.50 and shear rate $\dot{\gamma }/{\tau }^{-1}$ = 0, and two types of views are shown for various conditions.

**Figure 4:**
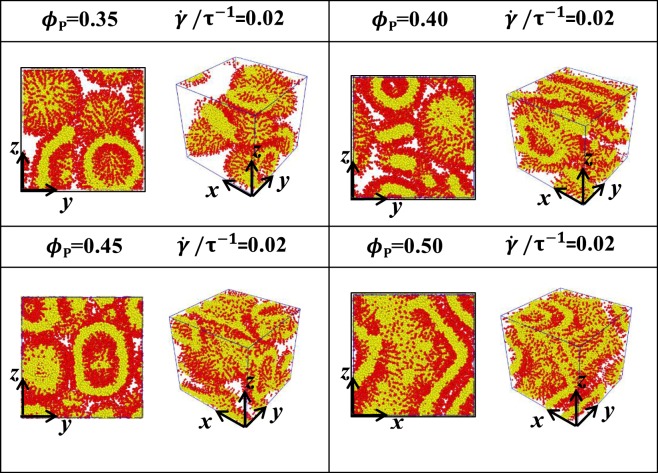
Microstructures of phospholipid pores with *N*_HB_ = 3 and *N*_TB_ = 4. The shear flows are applied perpendicular to the axes of pores, i.e., the z directions. The microstructures are arranged as functions of phospholipid concentrations *ϕ*_P_ = 0.35, 0.40, 0.45, 0.50 and shear rate $\dot{\gamma }/{\tau }^{-1}$ = 0.02, and two types of views are shown for various conditions.

**Figure 5:**
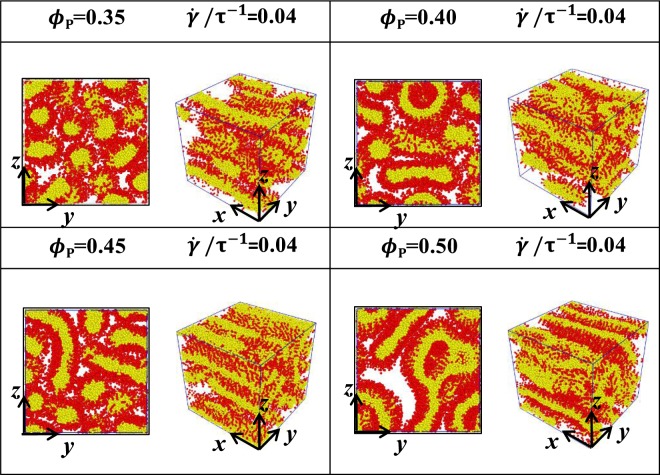
Microstructures of phospholipid pores with *N*_HB_ = 3 and *N*_TB_ = 4. The shear flows are applied perpendicular to the axes of pores, i.e., the z directions. The microstructures are arranged as functions of phospholipid concentrations *ϕ*_P_ = 0.35, 0.40, 0.45, 0.50 and shear rate $\dot{\gamma }/{\tau }^{-1}$ = 0.04, and two types of views are shown for various conditions.

**Figure 6:**
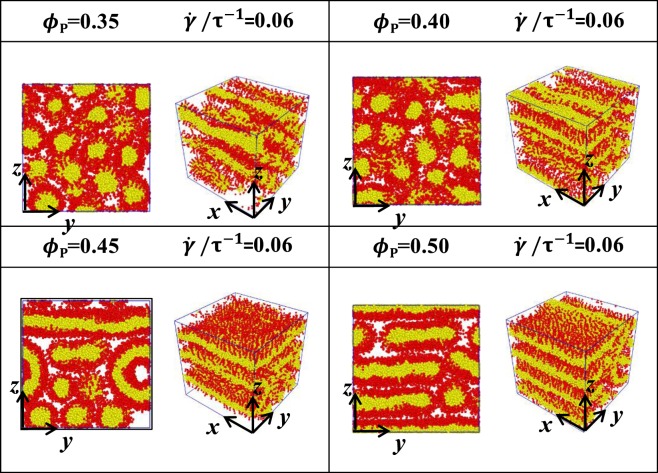
Microstructures of phospholipid pores with *N*_HB_ = 3 and *N*_TB_ = 4. The shear flows are applied perpendicular to the axes of pores, i.e., the z directions. The microstructures are arranged as functions of phospholipid concentrations *ϕ*_P_ = 0.35, 0.40, 0.45, 0.50 and shear rate $\dot{\gamma }/{\tau }^{-1}$ = 0.06, and two types of views are shown for various conditions.

**Figure 7:**
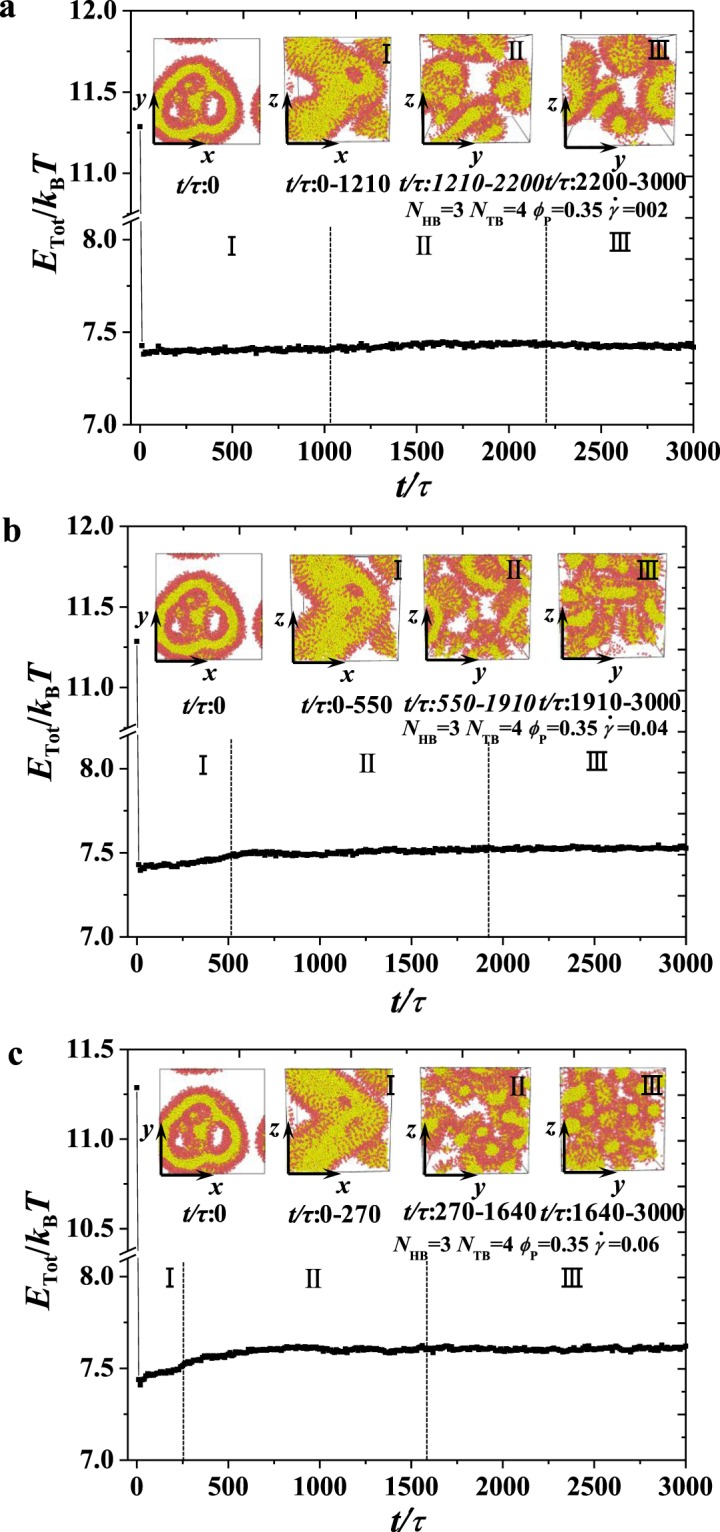
An example for obtaining the stable state with head bead numbers *N*_HB_ = 3, *N*_TB_ = 4 in the dynamic process under various shear rates at phospholipid concentration *ϕ*_P_ = 0.35.The total energy *E*_Tot_/*k*_B_*T* as function of the iteration step *N*_*s*_. The insets represent the microstructures of the system at the iteration steps of 0, 100000, 150000, and 300000, respectively. (**a–c**) for the total energy *E*_Tot_/*k*_B_*T* under shear rates $\dot{\gamma }/{\tau }^{-1}$ = 0.02, 0.04, and 0.06, respectively.

**Figure 8:**
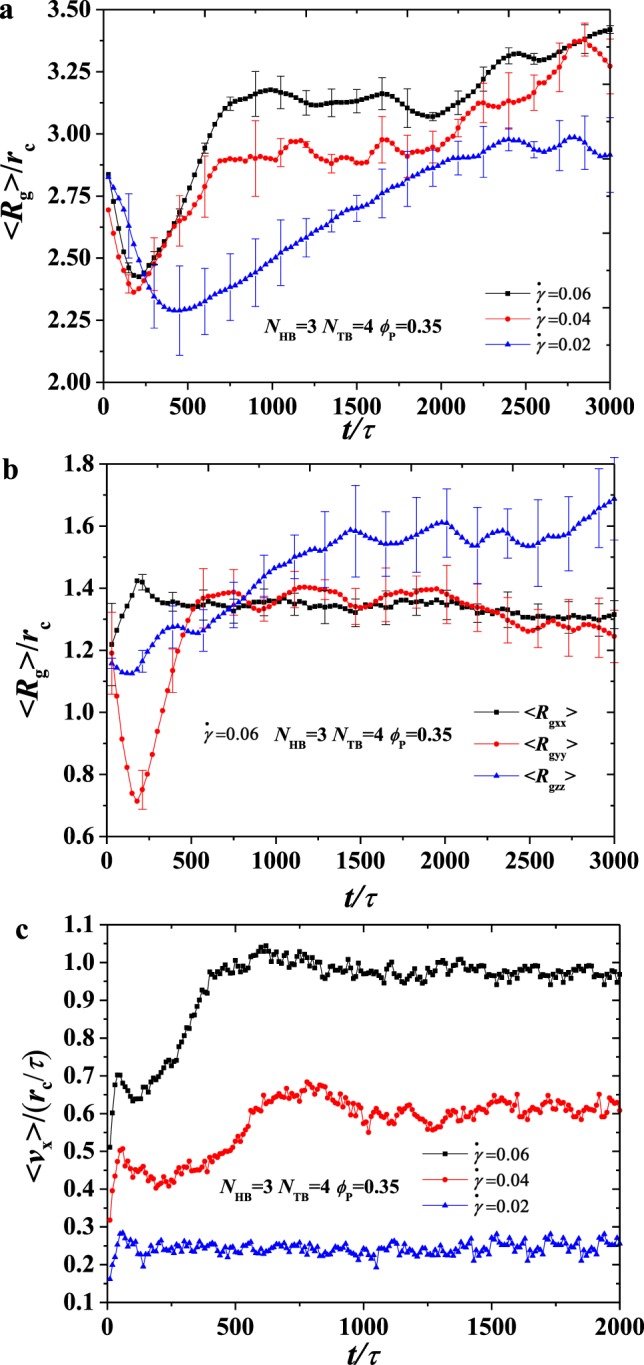
The average radius of gyration 〈*R*_*g*_〉 as functions of head bead numbers *N*_HB_ = 3, *N*_TB_ = 4 under various shear rates at phospholipid concentration *ϕ*_P_ = 0.35. (**a**) Different shear rates $\dot{\gamma }$. (**b**) The average radius of gyration in three direction. (**c**) An example of velocity profile in the simulation box with a selected set of parameter *N*_HB_ = 3, *N*_TB_ = 4 under different shear rates.

**Figure 9:**
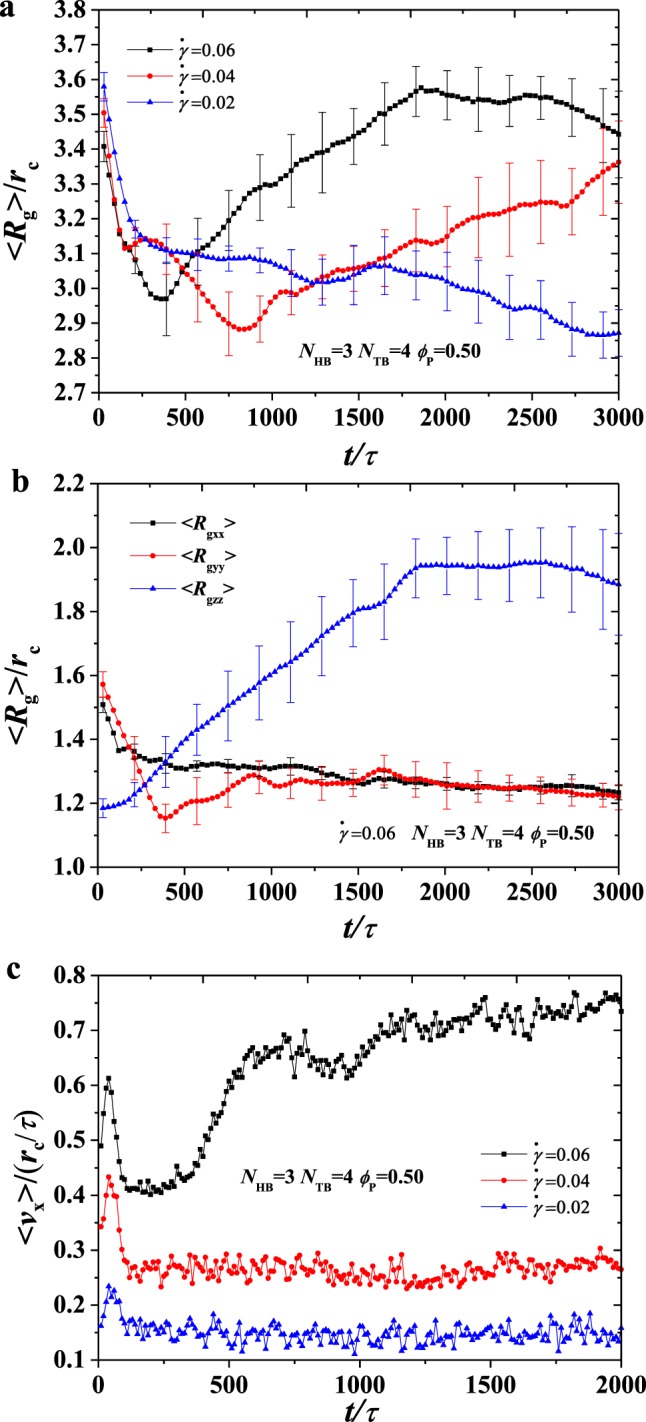
The average radius of gyration 〈*R*_*g*_〉 as functions of head bead numbers *N*_HB_ = 3, *N*_TB_ = 4 under various shear rates at phospholipid concentration *ϕ*_P_ = 0.50. (**a**) Different shear rates $\dot{\gamma }$. (**b**) The three parts of the average radius of gyration. (**c**) An example of velocity profile in the simulation box with a selected set of parameter *N*_HB_ = 3, *N*_TB_ = 4 under different shear rates.

**Figure 10:**
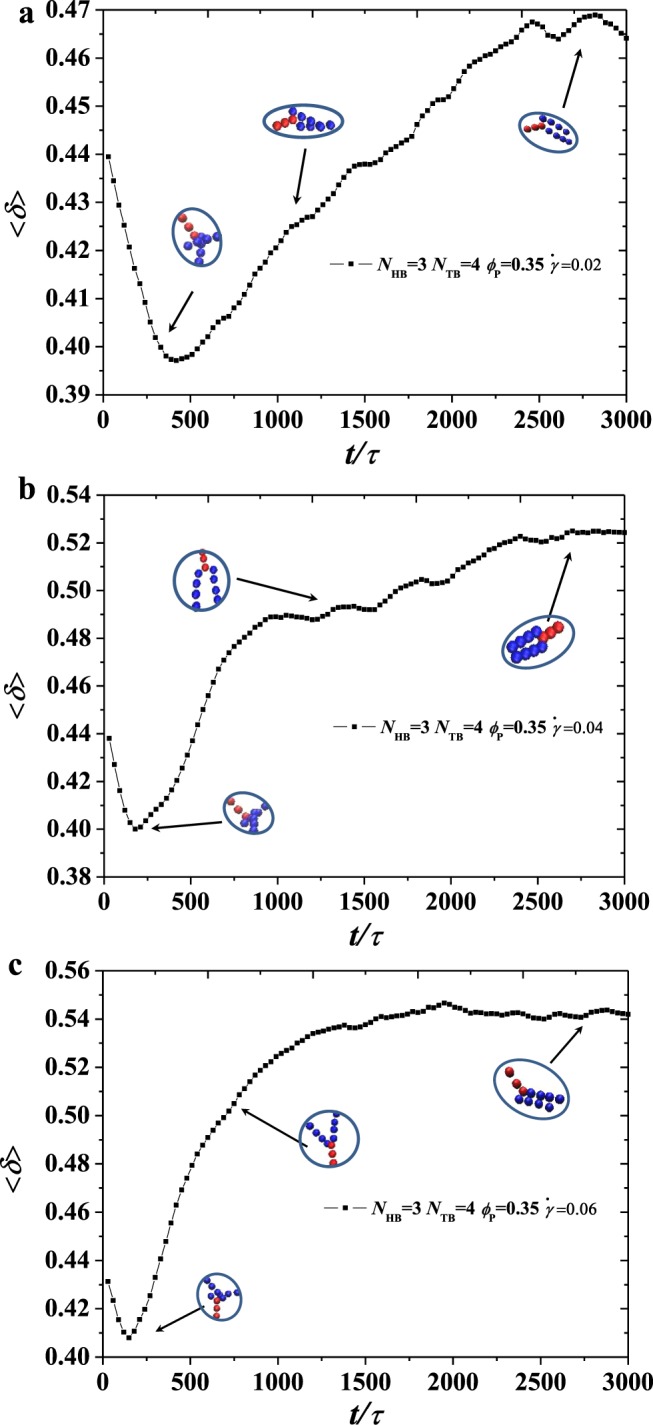
An example for obtaining the shape factor with head bead numbers *N*_HB_ = 3, *N*_TB_ = 4 in the dynamic process under various shear rates at phospholipid concentration *ϕ*_P_ = 0.35. The shape factor 〈*δ*〉 as function of the time *τ*. (**a–c**) for the shape factor 〈*δ*〉 under shear rates $\dot{\gamma }/{\tau }^{-1}$ = 0.02, 0.04, and 0.06, respectively.

We mainly focused on the microstructures of phospholipid porous cylinders with chain lengths of *N*_HB_ = 3 and *N*_TB_ = 4 under shear flows and focus on the various concentrations of *ϕ*_P_ = 0.35, 0.40, 0.45, 0.50 with the shear rates of $\dot{\gamma }/{\tau }^{-1}$ = 0.02, 0.04, 0.06. We discussed the equilibrium microstructures and the corresponding structural evolutions in Subsection 3.1. The obtained microstructures are shown in Figs 3–6, in which the head and tail beads are represented in red and yellow, respectively. The system energies in the dynamic processes are shown in Fig. 7, whilst the average radius of gyration and the shape factor with various time evolutions under different shear rates are presented in Figs 8–10. The structural evolutions are determined by analysing the average radius of the gyration and the shape factor in Subsection 3.2.

### Shear-induced microstructures

In this section, we studied the phospholipid porous cylinders with the various concentrations of *ϕ*_P_ = 0.35, 0.40, 0.45, 0.50 under the shear-free condition, as shown in Figs 3–6. Varying concentrations lead to a series of porous microstructures. In particular, several small cylinders are packed inside a large cylinder with distinct shapes of cross-sections and axes along the *z*-direction.

We observe porous cylinder structures with three small cylinders in a triangular arrangement at *ϕ*_P_ = 0.35, with four small cylinders in a quadrangular arrangement at *ϕ*_P_ = 0.40, with five small cylinders in pentagonal arrangement at *ϕ*_P_ = 0.45 and with six small cylinders in hexagonal arrangement at *ϕ*_P_ = 0.50, as shown in Fig. 3. In other words, the phospholipid concentration causes the small cylinders to be packed inside the large cylinder. This porous cylinder differs from the cylinder observed in the previous experiment and simulation, in which the cylinders are single or hexagonally arranged without porous cross-sections in the solutions^3,8^. Here, we observe phase transitions between the porous cylinders caused by the phospholipid concentrations.

The microstructures have weak shear rates of 0.02, as shown in Fig. 4. Considering that shear flow is applied perpendicularly onto the axes of the porous cylinder, structural transitions occur even under weak shear flows. In particular, the porous cylinders transit into several hollow vesicles at *ϕ*_P_ = 0.35, the mixtures of hollow vesicle and cylinders transit at *ϕ*_P_ = 0.40, 0.45 and distorted cylinders transit at *ϕ*_P_ = 0.50. Under weak shear flows, the shear force is insufficiently strong, but porous cylinders can still be broken up by the shear flow. The broken cylinders transit into the hollow vesicles at low concentrations. However, as the phospholipid concentration increases, the number of water molecules participating in the shear flow decreases. This condition leads to the weakening of the shear forces in the cylinders, whilst some cylinders maintain their original shapes, as shown in Fig. 4.

As shear flow increases to moderate strength, such as $\dot{\gamma }/{\tau }^{-1}$ = 0.04, the phospholipid cylinders transit into the broken lamellae mixed with the vesicles at the low *ϕ*_P_ values of 0.35 and 0.40, as shown in Fig. 5. At the high *ϕ*_P_ values of 0.45 and 0.50, the vesicles disappear, and the mixed structure is converted into irregular lamellae. This phenomenon is similar to the shear effects on the other system in which the micelles are elongated along the shear direction^42,58^. These irregular or broken lamellae have bilayered structures due to the amphiphilicity of the phospholipid molecules in the aqueous solutions^59^. The previous experiment has developed an array device to form the multilayer lipid cylinders under shear stress^60^, which differs from the presently reported mixed structures or lamellae but with similar bilayered structures.

For a strong $\dot{\gamma }/{\tau }^{-1}$ of 0.06, the vesicles develop into lamellae that connect the other parts and then form irregular lamellae in the entire system at the low *ϕ*_P_ values of 0.35 and 0.40, as shown in Fig. 6. At the high *ϕ*_P_ values of 0.45 and 0.50, a large number of phospholipid molecules participate in the assembly, and the irregular lamellae become planar structures, as shown in Fig. 6. These planar bilayer structures have various spatial separations because the phospholipid concentrations vary in the systems. The bilayer structures possess a large number of flat surfaces when *ϕ*_P_ increases. The shear-induced effects observed here are similar to the transition sequences in a lipid monolayer under shear flow by increasing shear rates^61^. However, shear flow does not affect the phospholipid cylinders by transiting into other structures but only sharpens their shapes along the pore directions regardless of shear rates (Fig. S2 in the Supplementary Materials). It is similar to those in applying a thin layer of plane shear, where the distance between the layers is more compact^23^.

### Dynamic processes

In this subsection, we focus on the dynamic process of the phospholipid microstructures. We also investigate the average radius of gyration and velocity of the phospholipid molecules with various concentrations and shear rates in these dynamic processes.

We initially concentrate on the dynamic processes of the formation of the hollow vesicle, broken lamella–vesicle mixture and irregular lamella at $\dot{\gamma }/{\tau }^{-1}$ = 0.02, 0.04, 0.06 for *ϕ*_P_ = 0.35, respectively. Then, we plot the system energies as functions of time step, as shown in Fig. 7. In the shear flow of $\dot{\gamma }/{\tau }^{-1}$ = 0.02 (Fig. 7a), the system spends approximately 2200 *τ* to equilibrate the state, and three stages are observed. The data show that 〈*E*_*Tot*_/*k*_*B*_*T*〉 = 7.602 exists in stable stages. In the first stage, the porous cylinder shape is distorted and pulled by shear forces, and the interactions between the phospholipid and water molecules play a basic role in gathering lipid molecules. The shape distortion stage is maintained at approximately 1050 *τ*, and the system evolves into a shape adjustment stage, in which the distorted PC-I is deformed. The adjustment stage is maintained approximately 1150 *τ*, as shown in Fig. 7a. The third stage is the vesicle formation stage, in which vesicles are formed, and the system enters an equilibrium state with stable energy. The dynamic processes in the system are similar to those observed in copolymer systems^13,58,62^. The previous experiments and simulations have reported the dynamic path of spherical vesicles for triblock copolymers in solutions by adding solvent rates^63^. Here, we report a distinct dynamic path from porous cylinders to hollow vesicles for phospholipid polymers in solutions by applying shear flows. Figure 7b,c show the formation processes of the broken lamella–vesicle mixture and the irregular lamella at $\dot{\gamma }/{\tau }^{-1}$ = 0.04, 0.06 for *ϕ*_P_ = 0.35, respectively. The dynamic processes are similar to those observed at $\dot{\gamma }/{\tau }^{-1}$ = 0.02 for *ϕ*_P_ = 0.35, in which the porous cylinder undergoes three stages in the entire process. However, the distortion stage is shortened as the shear rate is increased, and the system spends less time to converge into the formation stages with stable energy, as shown in Fig. 7b,c. This finding indicates that the shear effects on the dynamic processes can be achieved by shortening the convergence time.

We know that the radius of gyration tensor can more accurately characterize the structure and the square radius of gyration tensor ${{\bf{R}}}_{g}^{2}$ which is defined as follows^64^:

7 $${{\bf{R}}}_{g}^{2}=(\begin{array}{ccc}{R}_{gxx}^{2} & {R}_{gxy}^{2} & {R}_{gxz}^{2}\\ {R}_{gyx}^{2} & {R}_{gyy}^{2} & {R}_{gyz}^{2}\\ {R}_{gzx}^{2} & {R}_{gzy}^{2} & {R}_{gzz}^{2}\end{array})$$

where the elements ${R}_{g\alpha \beta }^{2}$ can be written as

8 $$\langle {R}_{{\rm{g}}\alpha \beta }^{2}\rangle =\frac{1}{N}\sum _{i}\langle ({r}_{i,\alpha }-{r}_{c,\alpha })({r}_{i,\beta }-{r}_{c,\beta })\rangle $$

with $\alpha ,\beta \in \{x,y,z\}$, *N* and *r*_*i,x*_ are the number of chains and *x*-coordinate of *i*th bead, respectively, whilst ***r***_*c*_ is the mass centre. Based on the radius of gyration tensor, we first discussed the average radius of gyration 〈*R*_*g*_〉 for the structure, which is defined as follow:

9 $$\langle {R}_{g}^{2}\rangle =\frac{1}{N}\mathop{\sum }\limits_{i=1}^{N}\langle {({{\bf{r}}}_{i}-{{\bf{r}}}_{c})}^{2}\rangle $$

so that we investigate the average radius of gyration and velocity for the phospholipid molecules with various concentrations and shear rates in dynamic processes, as shown in Figs 8 and 9. We plot the average radius of gyration 〈*R*_*g*_〉 for *ϕ*_P_ = 0.35 at various shear flows, as shown in Fig. 8a. The shear rate $\dot{\gamma }/{\tau }^{-1}$ = 0.0 is also shown in Fig. S3 in the Supplementary Materials. In general, 〈*R*_*g*_〉 strongly fluctuates in the dynamic processes at $\dot{\gamma }/{\tau }^{-1}$ = 0.02, 0.04, 0.06. This condition is probably due to the instability of the microstructure in the shear flows because of the movement of cylinders driven by the shear forces; this phenomenon is the same as the behaviour observed in the phospholipid vesicle under shear flows^28,32^. Indeed, the porous cylinder structures transit into the hollow vesicle at $\dot{\gamma }/{\tau }^{-1}$ = 0.02, in which the shear flows strongly affect their shapes. Porous cylinder transits into the broken lamella–vesicle mixture at $\dot{\gamma }/{\tau }^{-1}$ = 0.04 and into irregular lamella at $\dot{\gamma }/{\tau }^{-1}$ = 0.06, in which the lamellar structure has relative stability under shear flows. As a result, fluctuations cannot be easily observed in the strong shear flows. However, the differences between the average radius of gyration 〈*R*_*g*_〉 over the entire process are evident due to strong shear effects. Particularly, 〈*R*_*g*_〉 = 2.94 *r*_c_, 3.19 *r*_c_, 3.38 *r*_c_, as obtained by the linear fittings, which indicate the occurrence of shear effects under strong shear flows. In demonstrating the shear effect clearly, we plot the average radius of the gyration diagonal components in the dynamic processes for *ϕ*_P_ = 0.35 and $\dot{\gamma }/{\tau }^{-1}$ = 0.06, as shown in Fig. 8b. Three components, namely, 〈*R*_gxx_〉, 〈*R*_gyy_〉 and 〈*R*_gzz_〉, fluctuate strongly; however, 〈*R*_gzz_〉 is larger than 〈*R*_gxx_〉 and 〈*R*_gyy_〉, and 〈*R*_gxx_〉 is nearly equal to 〈*R*_gyy_〉. This result indicates nearly isotropic conformation distributions in the x–y plane in the irregular lamellae. However, the shear force elongates the chains along the z-direction. As a result, 〈*R*_gzz_〉 is considerably larger than 〈*R*_gxx_〉 and 〈*R*_gyy_〉. Therefore, we plot 〈*ν*_*x*_〉 as a function of time at *ϕ*_P_ = 0.35 under $\dot{\gamma }/{\tau }^{-1}$ = 0.02, 0.04, 0.06, as shown in Fig. 8c. 〈*ν*_*x*_〉 increases with time firstly and then elevates into the saturated values of 0.244, 0.605 and 0.973 at $\dot{\gamma }/{\tau }^{-1}$ = 0.02, 0.04, 0.06. Evidently, a large shear flow results in a large saturated velocity.

In comparing the effects of phospholipid molecular concentration and shear rate on self-assembled structures, we also plot the average radius of gyration 〈*R*_g_〉 for *ϕ*_P_ = 0.50 at various shear flows, as shown in Fig. 9a. The shear rate $\dot{\gamma }/{\tau }^{-1}$ = 0.0 is also shown in Fig. S4 in the Supplementary Materials. In general, 〈*R*_g_〉 strongly fluctuates in the dynamic processes at $\dot{\gamma }/{\tau }^{-1}$ = 0.02, 0.04, 0.06, which is consistent with our previous analysis. 〈*R*_g_〉 = 2.95 *r*_*c*_, 3.25 *r*_*c*_, 3.55 *r*_*c*_ are also obtained by the linear fittings, which obviously exhibit shear effects under strong shear flows. Compared with the concentration of 0.35, the average radius of gyration 〈*R*_g_〉 has significantly increased because the concentration of the phospholipids is higher, more phospholipid molecules participate in the self-assembly and the average radius of gyration increases with concentration. We also studied the three components of the average radius of gyration in the dynamic process with $\dot{\gamma }/{\tau }^{-1}$ = 0.06, as shown in Fig. 9b. 〈*R*_gzz_〉 is larger than 〈*R*_gxx_〉 and 〈*R*_gyy_〉^,^ and 〈*R*_gxx_〉 is nearly equal to 〈*R*_gyy_〉. Thus, we can know from the above results that strong shear flows can induce the crystallisation of phospholipid polymers; this phenomenon is consistent with what we observed for other polymers in shear flows in the literature^62^. Finally, we plot 〈*ν*_*x*_〉 as a function of time at *ϕ*_P_ = 0.50 under $\dot{\gamma }/{\tau }^{-1}$ = 0.02, 0.04, 0.06, as shown in Fig. 9c. The trend of the velocity distribution at the concentration of 0.5 is the same as the trend at the concentration of 0.35. 〈*ν*_*x*_〉 increases with time firstly and then elevates into the saturated values of 0.155, 0.27 and 0.74 at $\dot{\gamma }/{\tau }^{-1}$ = 0.02, 0.04, 0.06. However, the value at 0.5 concentration is significantly less than the value at 0.35, considering that shear flow is applied into the water molecules, and a high concentration leads to a small number of water molecules, thus resulting in low saturated velocities.

The self-assembled structural changes in the applied flow field can be derived from Figs 8 and 9. In comprehensively describing the information of the polymer chains constituting the structure, we calculate the shape factor derived from the square radius of gyration tensor ${{\bf{R}}}_{g}^{2}$, which can be expressed as follows^65,66^:

10 $$\langle \delta \rangle =1-3\langle \frac{{L}_{1}^{2}{L}_{2}^{2}+{L}_{2}^{2}{L}_{3}^{2}+{L}_{1}^{2}{L}_{3}^{2}}{{({L}_{1}^{2}+{L}_{2}^{2}+{L}_{3}^{2})}^{2}}\rangle $$

where ${L}_{1}^{2}$, ${L}_{2}^{2}$ and ${L}_{3}^{2}$ are the three eigenvalues of the square radius of the gyration tensor ${{\bf{R}}}_{g}^{2}$. For convenience, we sort them by ${L}_{1}^{2}\le {L}_{2}^{2}\le {L}_{3}^{2}$. We plot the shape factor 〈*δ*〉 as functions of head bead numbers *N*_HB_ = 3, *N*_TB_ = 4 under various shear rates at the phospholipid concentration of *ϕ*_P_ = 0.35, as shown in Fig. 10.

The graph in Fig. 10 shows the relationship of the shape factor with simulation time at different shear rates. Shape factor 〈*δ*〉 decreases firstly and then increases as simulation time increases. According to the previous literature, when the shape factor 〈*δ*〉 is equal to 1 in 3D space, the structure tends to have a rod-like structure. When the shape factor 〈*δ*〉 is equal to 0, the polymer chain appears as a spherical structure; when the shape factor 〈*δ*〉 is equal to 0.5, it appears as a circular structure^67^. As shown in Fig. 10a, at the lowest point of shape factor 〈*δ*〉, the polymer chain of the system forms a state of cluster, which is close to the change of the ellipsoidal structure. This finding can be attributed to the tail end of the polymer chain, which has a certain flexibility. With the extension of simulation time, the polymer chain slowly expands under the action of shearing and finally tends to reach the stable state. The shape is changed to a circular shape given 〈*δ*〉 = 0.465. Moreover, in the case of weak shear at $\dot{\gamma }/{\tau }^{-1}$ = 0.02, a hollow vesicle structure is finally formed. Figure 10b shows that the same polymer chain remains to be agglomerated together in the case of moderate shear $\dot{\gamma }/{\tau }^{-1}$ = 0.04. The value of the shape factor 〈*δ*〉 increases gently with the increase of simulation time and finally becomes steady at the shape factor 〈*δ*〉 = 0.52. This finding indicates that in the medium shear state, in addition to the easy formation of a circular structure, the increase of shear force allows the structure of the polymer chain to expand more. Furthermore, the shape is likely an ellipse, which can be explained by $\dot{\gamma }/{\tau }^{-1}$ = 0.04, in which the structure is a mixed structure of hollow vesicles and broken lamellas. As shown Fig. 10c, the trend of the shape factor also decreases firstly and then increases and tends to be gentle. However, in the case of $\dot{\gamma }/{\tau }^{-1}$ = 0.06, the shape factor 〈*δ*〉 = 0.54 indicates that the polymer is under a strong shear rate. The structure of the chain tends to become more elliptical, which facilitates the formation of an irregular lamella.

By studying the shape factor 〈*δ*〉 under three shear strengths for the concentration of *ϕ*_P_ = 0.35, we can clearly see that as the shear strength increases, the shape factor 〈*δ*〉 gradually becomes larger, and the structure of the polymer chain is changed from the previous ellipse. Moreover, as the shear rate increases, the shape factor becomes gradually larger at the lowest point. This finding can be attributed to the shear factor that causes the shape factor to operate similar to a circular transition. The spherical shape develops towards the ellipse, and the increase of the shear rate also leads to the change in the rate of the polymer structure. In the strong shear state, the polymer chain can rapidly change from an ellipsoid state to an elliptical state. This finding indicates that the applied shear rate can accelerate the change in shape of the polymer chain.

## Conclusions

We investigated the shear-induced assembly of phospholipid microstructures, by using DPD simulation based on the CG model in water solutions. We selected the shear-free phospholipid cylinders with various cross-sectional shapes and subsequently observed their shear-induced microstructures and the corresponding dynamic processes at various concentrations by changing $\dot{\gamma }/{\tau }^{-1}$. We observed the vesicles, lamellae and their mixtures in the water solutions at various *ϕ*_P_ and shear rates. The results show that phospholipid molecules form a lamellar structure under strong shear flows, whereas a mixture of vesicle and lamellae can be observed under moderate shear flows. However, weak shear flows easily form phospholipid vesicles in the solutions. Even if the shear rate is enhanced, only the phospholipid porous cylinders can be observed to become a lamellar structure more quickly, and it is necessary to observe the new structure by changing the model. Extending the simulation time does not change the results.

The dynamic processes indicate that porous cylinders undergo three stages, namely, distortion, adjustment and formation, in the entire dynamic process. Strong shear flows can shorten the distortion and adjustment stages, thereby resulting in the rapid progress into the formation stage. The average radius of gyration and velocity in the dynamic processes indicates that shear rate plays an important role in pulling phospholipid molecules into the solutions under various concentrations. In obtaining more information about the polymer, we also studied the shape factor 〈*δ*〉 of the polymer chain. The results show that the shape factor 〈*δ*〉 increases with the increase of simulation time and finally becomes flat. Under different shear rates, the shape factor 〈*δ*〉 also increases with the increase of shear strength. According to the trend of the shape factor 〈*δ*〉 under the action of strong shears, the polymer structure can quickly change its initial structure to an ellipsoidal structure, and when the shear force increases, the shape factor 〈*δ*〉 of the polymer chain is also increased. This work offers insights into the shear-induced microstructures and the dynamic processes of phospholipid cylinders.

## Supplementary information

Supplementary information

## Data Availability

The datasets generated during the current study are available from the corresponding author on reasonable request.
