# Supplementary material for: Shear-induced microstructures and dynamics processes of phospholipid cylinders in solutions
Source: Sci Rep. 2019 Oct 28;9:15393. doi: 10.1038/s41598-019-51933-z (PMC6817888; doi:10.1038/s41598-019-51933-z)
Supplement: Supplementary file 1 — Supplementary information [file 41598_2019_51933_MOESM1_ESM.pdf]

Shear-induced microstructures and dynamics processes of phospholipid cylinders in solutions

Yue Shan[1], Xiaowei Qiang[1], Jianzhu Ye[1], Xianghong Wang[2], Linli He[1] and Shiben Li[1,*]

[1]*Department of Physics, Wenzhou University, Wenzhou 325035, Zhejiang, China;*

[2]*Department of Physics, Wenzhou Vocational & Technical College, Wenzhou 325035, Zhejiang, China;*

[*]Corresponding author: Shiben Li (E-mail: shibenli@wzu.edu.cn)

Fig.S1

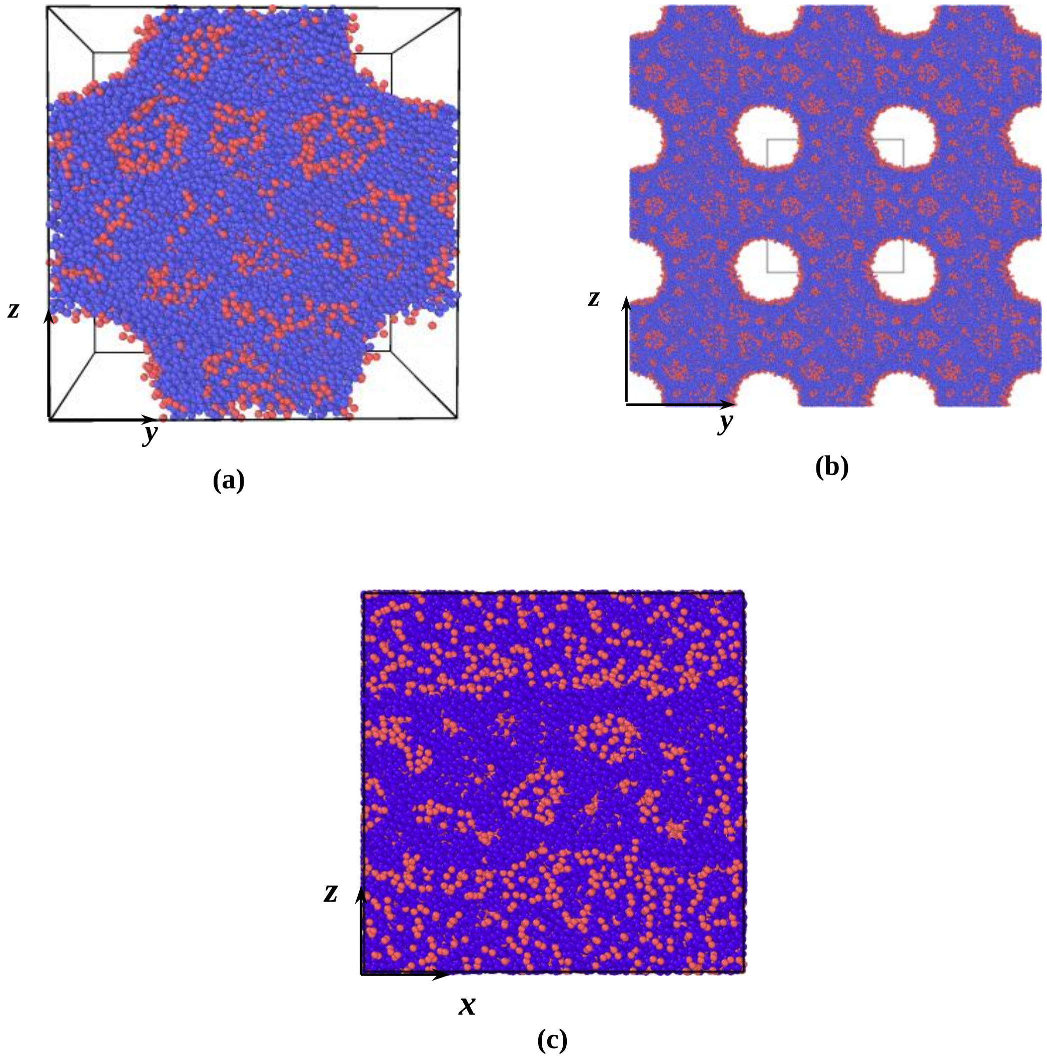

Caption of Fig. S1

The microstructure of the cylindrical network structure from different perspectives. (a) Side view of the microstructure. (b) Side view of the microstructure under periodicity. **(**c) Side view of the microstructure.

Fig. S2

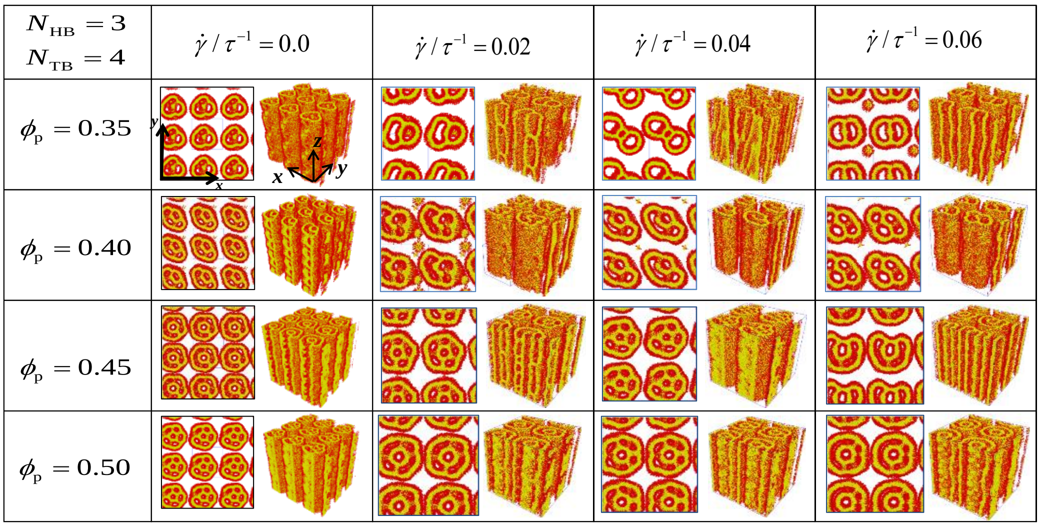

Caption of Fig. S2

Microstructures of phospholipid porous-pores with N_{HB}=3 and N_{TB}=4. The shear flows are applied parallel to the axes of pores, along with the z direction. The microstructures are arranged as functions of phospholipid concentrations ϕ_P and shear rate $\dot{\gamma}$, and two types of views are shown for various conditions.

Fig. S3

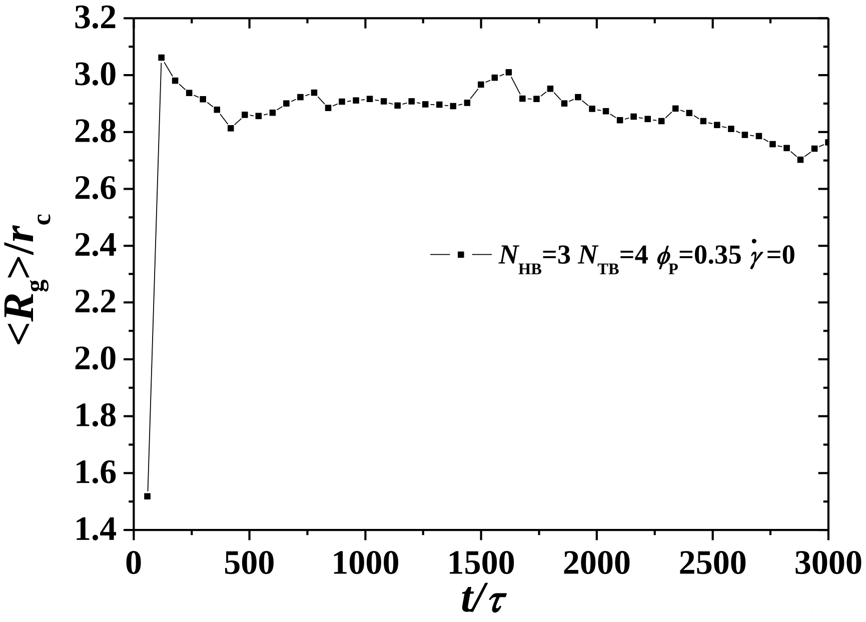

Caption of Fig. S3

The average radius of gyration $\langle R_g \rangle$ as functions of time step τ at parameters of N_{HB}=3, N_{TB}=4 and ϕ_P=0.35.

Fig. S4

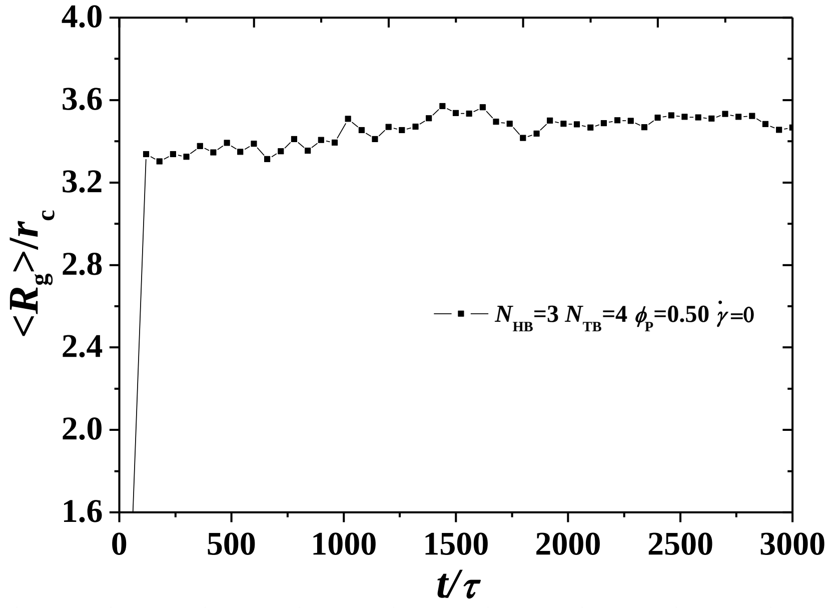

Caption of Fig. S4

The average radius of gyration $\langle R_g \rangle$ as functions of time step τ at parameters of N_{HB}=3, N_{TB}=4 and ϕ_{P}=0.50.
